# Myc and the Replicative CMG Helicase: The Creation and Destruction of Cancer

**DOI:** 10.1002/bies.201900218

**Published:** 2020-02-20

**Authors:** Damon R. Reed, Mark G. Alexandrow

**Affiliations:** Department of Interdisciplinary Cancer Management, Moffitt Cancer Center and Research Institute Tampa, FL 33612, USA; Department of Molecular Oncology, Moffitt Cancer Center and Research Institute, Tampa FL 33612, USA

**Keywords:** Cdc45-MCM-GINS helicase, chemotherapy, DNA replication, Myc, tumorigenesis

## Abstract

Myc-driven tumorigenesis involves a non-transcriptional role for Myc in over-activating replicative Cdc45-MCM-GINS (CMG) helicases. Excessive stimulation of CMG helicases by Myc mismanages CMG function by diminishing the number of reserve CMGs necessary for fidelity of DNA replication and recovery from replicative stresses. One potential outcome of these events is the creation of DNA damage that alters genomic structure/function, thereby acting as a driver for tumorigenesis and tumor heterogeneity. Intriguingly, another potential outcome of this Myc-induced CMG helicase over-activation is the creation of a vulnerability in cancer whereby tumor cells specifically lack enough unused reserve CMG helicases to recover from fork-stalling drugs commonly used in chemotherapy. This review provides molecular and clinical support for this provocative hypothesis that excessive activation of CMG helicases by Myc may not only drive tumorigenesis, but also confer an exploitable “reserve CMG helicase vulnerability” that supports developing innovative CMG-focused therapeutic approaches for cancer management.

## Graphical Abstract Legend

Myc is involved in the conversion of MCM complexes into CMG helicases for DNA replication, but too much Myc over-activates CMG helicases. This reduces the number of unused Reserve CMGs necessary for recovery from replicative stresses, promoting tumorigenesis but also creating an exploitable CMG weakness in tumor versus normal cells.

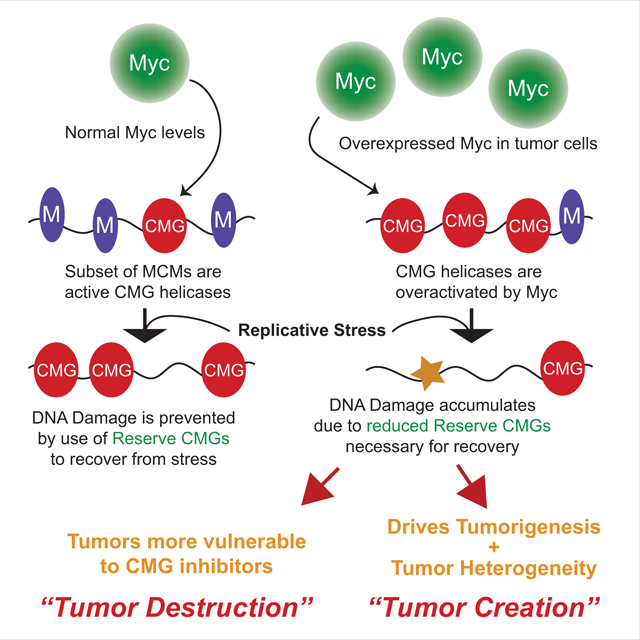

## 1. Introduction

The development of cancer is a multistep process involving, in part, the acquisition or inheritance of mutations in oncogenes that renders their oncoprotein products overactive due to regulatory changes, gain of function mutations, or overexpressed conditions. Tumors are often dependent on these deregulated oncoproteins for development and maintenance, leading to the conventional definition of these oncoproteins as drivers of tumorigenesis. Examples of oncogenic drivers include mutation or deregulated expression of genes such as *EGFR*, *Src*, *Ras*, *Raf*, *Myc*, and others. Whereas such drivers promote carcinogenesis due to their ability to stimulate proliferative capacity of tumor cells, other drivers of cancer include proteins/enzymes whose decreased function creates a tumor-conducive environment within cells. These tumor suppressors are also typically rendered less active through inactivation or loss. Examples of the latter include loss of DNA replication fidelity due to mutation of *MutS* homolog genes, abrogation of apoptosis due to *Bcl2* mutations, or loss of function mutations in tumor suppressor genes *Tp53* and *RB1*. Mutation or deregulated expression of these drivers of tumorigenesis can be detected in cancers using next generation sequencing (NGS), exome sequencing, or genome wide association studies (GWAS). In contrast to these drivers, the majority of genes are frequently characterized as “housekeeping” and rarely or never mutated, nor included in targeted sequencing panels clinically.

Emerging evidence suggests that the replicative CMG (Cdc45MCM-GINS, described in more detail below) helicase may also be a driver of tumorigenesis despite it being in this last category of never-mutated genes. Many studies have found that tumor tissue and biopsy samples display elevated expression of CMG components relative to non-tumor tissue.^[1,2]^ Such elevated expression of CMG components in tumor tissue is often referred to as “overexpressed,” providing an argument that CMG helicases (like other overexpressed cell cycle regulating proteins) might be drivers of cancer growth and thus suitable for the development of drugs that can inhibit CMG function for cancer treatment (reviewed in ref. [1]). However, in terms of protein stoichiometries/numbers, there is little evidence that the CMG protein components are actually overexpressed or under-expressed in individual tumor cells relative to their stoichiometries in non-tumor proliferating cells. Quite the opposite, there is quantitative evidence that CMG protein components are expressed at very similar levels (in terms of total protein molecules) in individual proliferating non-tumor and tumor cells.^[3]^ A likely explanation for these discrepancies is that tumor tissue is in a heightened proliferative state that renders the CMG helicases noticeably elevated in immunohistochemical staining assays relative to that seen in normal, non-tumor tissue with a lower proliferative index (or non-proliferating tissue).

Consistent with CMGs not necessarily being overexpressed drivers of cancers, NGS, GWAS, and exome sequencing approaches tend not to identify alterations in CMG expression or gene integrity associated with human cancers, indicating that the genes coding for CMG subunits are not amplified or mutated in human cancers. For example, a search of more than 42 000 human tumor samples in cBioPortal (www.cbioportal.org; accessed August 2, 2019) reveals that none of the 11 genes coding for CMG subunits contain hotspot mutations or amplifications beyond a basal background of overall genomic mutation rates. Collectively, these observations would argue that the CMG helicase and its components cannot be easily defined as overexpressed or mutated drivers of carcinogenesis as is often the case for many oncogenic proteins. However, understanding the biochemistry and cell biology of CMG helicase assembly and activation, and how this process is mismanaged by oncoproteins such as c-Myc and Cyclin E, produces a situation where the CMG helicase may well be a driver of carcinogenesis that is suitable for drug development and clinical intervention efforts.

**Figure 1.**
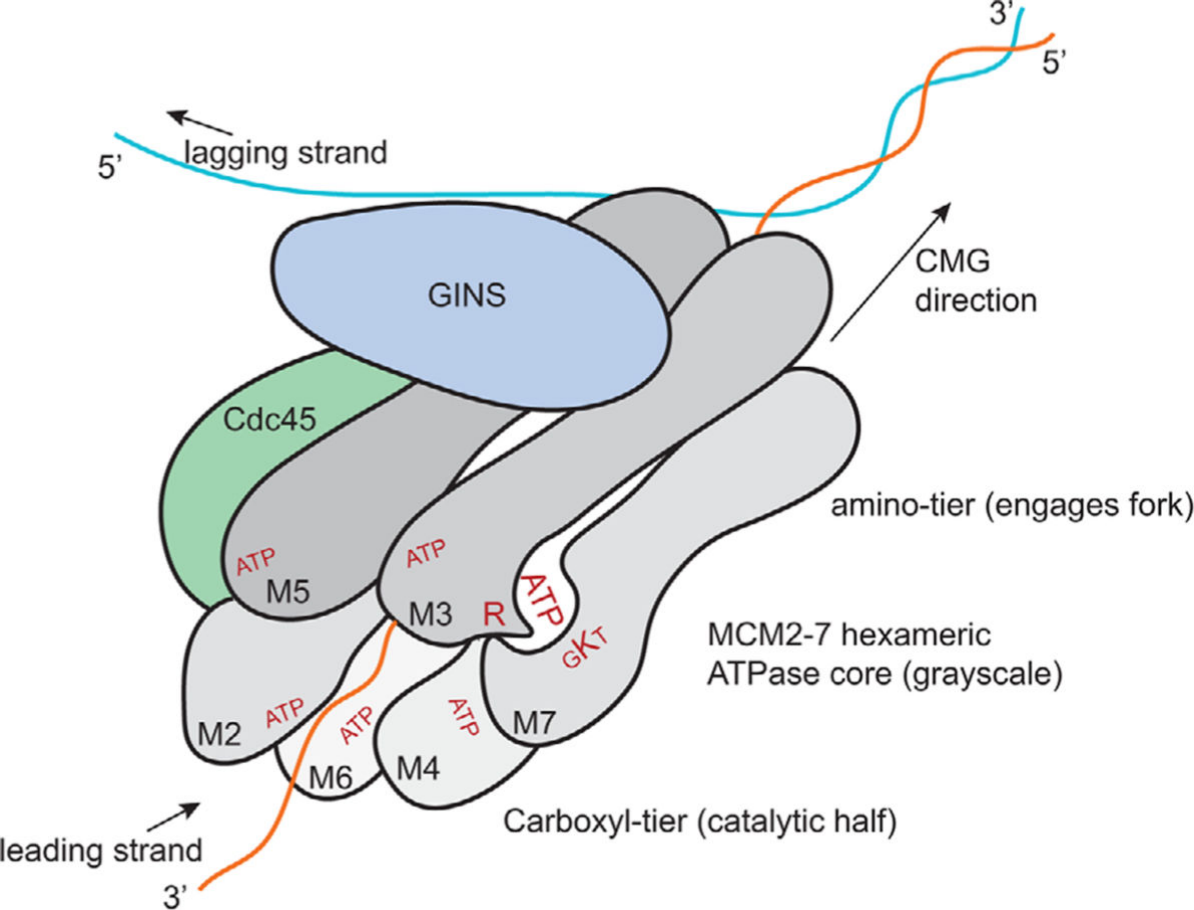
Structure and ATP clefts of the CMG helicase (M2–7 are the MCM2–7 subunits, and more details are shown for the Mcm3–7 cleft;).

The replicative CMG helicase is an 11 subunit enzyme that catalyzes the melting of DNA during DNA replication and is required for resuming DNA replication during recovery from replicative stresses.^[4–8]^ CMG subunits include Cdc45, the MCM2–7 hexamer (mini-chromosome maintenance), and a GINS tetramer (Go-Ichi-Ni-San) comprising Sld5 and Psf1–3^[6,7,9]^ (Figure 1). Multiple steps are involved in regulating CMG assembly and activation in eukaryotic cells, and elegant molecular and structural descriptions of this process are covered in several reports.^[5,6,10–12]^ Inbrief, the CMG assembly steps can be assigned to two stages. In Stage-1 of assembly, the MCM2–7 hexameric ATPase core of the helicase is loaded onto DNA in excess during G1 phase, as a head-to-head set of double hexamers averaging ≈4–6 MCM hexamers per 100 kb in mammalian cells (i.e., 2–3 hexamer pairs per 100kb).^[3,10,11,13]^ This serves two purposes. First, the MCMs mark chromosomes that have not yet replicated, and thus license such chromosomes for precisely one round of DNA replication.^[9,14]^ Second, a subset of the MCMs that have licensed non-replicated chromosomal regions will become sites of future origins of DNA replication.^[15,16]^ At Stage-2, which occurs in lateG1 or at the G1-S transition, Cdc45 and GINS are recruited to this subset of MCMs to form CMG helicases (as a pair) that will function in initiation and bidirectional elongation of DNA replication within replisomes.^[4,6,7,17]^ As will be described in more detail below, emerging evidence has shown that in mammalian cells the c-Myc protein is required for and promotes the recruitment of Cdc45 and GINS to pre-existing MCM hexamers.^[18–20]^ This role for c-Myc in CMG assembly and activation has the propensity to confer a driver function on CMGs during tumorigenesis, while simultaneously creating an exploitable vulnerability in CMG function in cancer cells that may provide a novel therapeutic target for cancer intervention.

## 2. Mismanagement of MCMs/CMGs as a Cancer Driver?

### 2.1. Reserve MCMs/CMGs: Necessary for Recovery from Replicative Stress

A key for understanding how MCM/CMG mismanagement can perform a driver function for tumorigenesis, and as described later, an exploitable weakness in tumors comes from evidence that not all excessively loaded MCMs appear to be converted to CMGs during DNA replication.^[16,21]^ Excess unused MCM hexamers loaded throughout the genome (not seemingly converted to a CMG), while involved in licensing non-replicated DNA, are also known as dormant origins.^[16,21,22]^ An interpretation of published results suggests that these reserve MCMs can become active CMG helicases during recovery from replicative stress.^[8,16,23]^ Numerically, mammalian cells contain an ≈five- to tenfold excess of MCMs than are required to complete an S-phase.^[3,8,16,23]^ Multiple studies have found that experimental depletion of the excess MCMs, while not blocking DNA replication itself, leads to sensitivity of cells to replicative stresses induced by exposure to replication fork-stalling drugs such as hydroxyurea and aphidicolin.^[8,16,23]^ Predictably, further depletion beyond the reserve MCMs, reducing the MCMs minimally required for DNA replication to start and finish, halts S-phase progression.^[8,16,23]^ It should be noted, though, that the actual fate of reserve MCMs at dormant origins in terms of their conversion to CMGs during ongoing DNA replication (but remaining inactive), or conversion only after replicative stress, has not been conclusively determined. Experimentally, a genomic chromatin-crosslinking, immunoprecipitation and sequencing (ChIP-seq) based approach analyzing multiple CMG subunits would be necessary to ascertain the degree to which MCMs are converted to CMGs before or during DNA replication, or after replicative stress, to give a clear picture of MCM-to-CMG dynamics. However, regardless of such molecular specifics, the excess reserve MCMs (or CMGs) are clearly necessary for efficient recovery from replicative stresses.

### 2.2. Human Tumor Cells Need Reserve MCMs for Chemotherapy Recovery

Of clinical relevance, reduction of the reserve complement of MCMs renders pancreatic ductal adenocarcinoma (PDAC) and colorectal carcinoma (CRC) cells sensitive to chemotherapeutic drugs commonly used to treat such cancers, in this case 5-fluorouracil, gemcitabine, and etoposide.^[8]^ Importantly, these drugs are fork-slowing drugs that induce replicative stress on the tumor cells. Under these conditions of stalled replication forks, cells attempt to recover DNA replication (after any repairs) using the reserve MCMs as substrates to form a new CMG helicase and replicate remaining DNA regions.^[8,16,23]^ The previously functioning CMGs are seemingly not used (or stalled), and may be disassembled, although the fate of such previous CMGs remains poorly understood. Failure to utilize reserve MCMs for recovery yields DNA damage signaling responses and genomic instability.^[16,21,23]^ Thus, during replicative stress, the dormant unused reserve MCMs are necessary for recovery of fork progression, thus ensuring fidelity of DNA replication and genomic stability.

### 2.3. Weakened MCM Reserves Drive Tumorigenesis in Mice

A clear demonstration of how mismanagement of reserve MCM complexes can produce a tumor-driver effect has been shown in an elegant set of studies using a transgenic mouse model in which MCM hexamers are destabilized due to a specific acquired mutation in the *Mcm4* gene.^[21,24]^ The mutation in *Mcm4*, referred to as the *Mcm4*^*Chaos3*^ allele, does not result in a loss of CMG helicase enzymatic activity (as measured in vitro), but rather causes a destabilization of MCM hexamer binding to chromatin throughout the genome such that the entire cellular complement of MCM hexamers, including reserves, is functionally weakened.^[21,24]^ Fibroblasts obtained from these mice show increased numbers of stalled replication forks, DNA damage signals, and activation of fork recovery events, even though DNA replication has not been perturbed by any external means such as fork inhibitors.^[21]^ Intriguingly, these problems in S-phase seem to be somewhat accepted by the cells, perhaps at a low level, and remain unfixed as cells enter M-phase.^[21]^ This leads to problems during chromosome segregation and an acquisition of genomic deficiencies.^[21]^ Although difficult to prove directly in these studies, it appears likely that these genomic problems promote tumorigenesis, as the mice carrying this mutant *Mcm4* allele are tumor prone without additional tumor promoters, initiators, or interbreeding with other oncogene-carrying mice being needed.^[21]^ Depending on the mouse strain analyzed, breast adenocarcinomas, histiocytic sarcomas, or lymphomas arise.^[21,24,25]^ In other studies, weakened dormant MCMs due to mutation of *Mcm2* produce thymomas, lung carcinomas, or liver carcinomas depending on mouse strains assessed.^[26,27]^ Thus, it may be that a natural selection occurs under conditions of globally weakened MCM reserves, and the concomitant accumulation of DNA damage from S-phase and M-phase malfunctioning, that leads to loss of growth control and eventual tumor formation.^[21,24–26]^ In this sense, proper reserve MCM management is tumor-suppressive, while reserve MCM mismanagement is tumor-promoting (or tumor-driving).

A similar situation arises for MCM loss in aging hematopoietic stem cells (HSC), which functionally decline in activity over time. Such HSC display elevated levels of replicative stress as a result of diminished MCM expression.^[28]^ The reduced MCM availability in cells, which would likely limit the reserve MCM complement, leads to replicative stress, changes in replication fork speed, DNA damage, and chromosome breaks that collectively contribute to the functional decline in HSC activity.^[28]^ Therefore, MCM reduction and consequent genomic instability can elicit biological effects separate from tumor promotion.

### 2.4. Why Are Human Malignancies Not Associated with Mutant MCMs?

Given that the *Mcm4* mutant mice are tumor prone, yet are not subjected to external stresses for DNA replication that stall forks, one might ask why there is evidence of stalled forks in such mouse cells.^[21]^ Even more relevant, one might also ask why human tumors do not seem to arise with underlying mutations in *Mcm* genes that weaken reserve MCM hexamers, display similar stalled forks, and produce genomic instability subjected to selection pressure at the cellular level that results in tumorigenesis. It is likely that normal DNA replication faces hurdles that create fork stalling situations, such as replication forks encountering heterochromatin, topological constraints of certain genomic regions, interference with transcriptional or related machinery, or impasses involving DNA lesions. These and other replicative stress conditions can be mitigated by cells using template switching, homologous recombination, fork reversal, or similar methods (for reviews, see refs. [29, 30]). Replicative stress situations may also require cessation of DNA replication from functioning CMGs, and then resumption of DNA replication from reserve MCMs/CMGs at dormant origins (for reviews, see refs. [30, 31]). Indeed, these fork stalling events would be equally present in mouse and human cells, thus suggesting that similarly mutated *Mcm* genes could promote human carcinogenesis as occurs in the mice. However, it appears that under certain circumstances mice with mutant *Mcm* genes die in utero, indicating that there can be developmental defects when *Mcm* genes are mutated and produce deficient MCM hexamers on a global level within all cells.^[21,26]^ In addition, the same engineered mutation in *Mcm4* produces a mini-chromosome loss phenotype in yeast, indicating that overall DNA replication can be adversely affected insome eukaryotes.^[21,25–27]^ It may be that for similar reasons human cells do not tolerate global deficiencies of MCM mismanagement on a cellular or developmental level, or that human cells have more complex mechanisms of sensing reserve MCM mismanagement. Such reasons could explain the lack of *Mcm* gene mutations observed in human malignancies, either inherited or acquired in somatic cells, which would affect the entire complement of MCM hexamers at the cellular or organismal level.

## 3. Overexpression of the c-Myc Oncoprotein Mismanages Reserve MCMs/CMGs

Although human tumors have not yet been found to be driven by *Mcm* mutations that adversely affect reserve MCM management, there is evidence that certain oncogene products do affect reserve MCM pools and CMG activation dynamics. The assembly of the CMG at pre-established MCM hexamer sites requires the function of the c-Myc protein (referred to hereafter as Myc), specifically in late-G1 when Cdc45 and GINS are recruited onto MCMs in mammalian cells.^[20]^ Myc interacts with many genomic sites, including promoter regions and intergenic regions, the latter of which are also regions where MCM hexamers are loaded onto DNA.^[32,33]^ The genomic proximity of Myc to MCM complexes is associated with a function for Myc in recruiting the GCN5 and Tip60 histone acetyl-transferases (HATs), which cause chromatin decondensation necessary for Cdc45/GINS recruitment to MCMs^[20]^ (for details, see Box 1). Myc forms complexes with Cdc45 and MCMs,^[19,20]^ although it remains to be determined experimentally whether Myc makes direct contacts with these subunits or the CMGs themselves. The absence of Myc or such HAT activity in late-G1 prevents CMG formation and S-phase entry.^[20]^ Importantly, this role for Myc in regulating CMG assembly and activation is independent of the conventional functions for Myc in transcription.^[18–20]^

This molecular mechanism for Myc in regulating CMG assembly has important implications for tumorigenesis driven by Myc. It is estimated that ≈70% or more of human cancers have Myc overexpressed or deregulated in other ways that lead to elevated Myc activity in tumor cells.^[34–36,37]^ Elevated Myc protein is known to expand its interactions within the genome,^[33,38]^ and presumably this would be met with increased interactions with MCMs/CMGs. Although formal demonstration of elevated MycCMG interactions in tumor cells versus non-tumor cells has not been shown experimentally, but could be addressed using ChIPseq based approaches, it is known from several studies that overexpression of Myc does indeed produce an increase in replication origin usage and CMG helicase over-activation,^[18–20]^ consistent with the mechanisms underlying Myc stimulation of CMG assembly. These findings suggest that, in addition to transcriptional control by Myc, another influence of Myc during tumorigenesis may be the over-activation of CMG helicases, which would necessarily occur due to over-activation (mismanagement) of the reserve pool of MCMs/CMGs. This concept is also nicely discussed in a review on Myc control of DNA replication.^[39]^

This ability of overexpressed Myc, a tumor-driver itself, to overactivate and mismanage MCMs/CMGs has the propensity to create a new tumor-driver situation with regard to the reserve MCM capacity. Although conceptually different from the reduced reserve capacity of MCMs in the *Mcm4*^*Chaos3*^ mutant mice with weakened MCM-chromatin interactions,^[21]^ over-activation of reserve MCMs by Myc results in a similar functional outcome where the number of unused reserve MCMs are reduced due to Myc’s actions.^[18–20]^ The reduction in unused reserve MCM capacity by Myc overexpression renders cells less effective at handling stalled replication forks.^[18,19,21]^ Similar to that seen with the *Mcm4*^*Chaos3*^ mouse model, a Myc-driven deficiency in reserve MCM capacity concurrent with normal problems in Sphase that produce stalled replication forks will likely create an unstable genomic environment and DNA damage accumulation that leads to positively selected adaptations in mutated cells and tumorigenesis.^[21,24]^ Since Myc is sometimes overexpressed in early stages of tumorigenesis,^[35,40]^ it is possible that deregulation of Myc in benign tumors or early-stage cancers leads to genomic instability issues arising from reduced reserve MCM capacity, which facilitates selection and progression to later neoplastic stages. Indeed, Myc overexpression is known to be associated with acute onset of genomic instability.^[18,41,42]^ producing replication stress, fork rate slowing, and fork asymmetry in a single cell cycle in a human cancer cell analysis.^[42]^ MCM/CMG over-use by Myc may be one underlying molecular explanation for these effects.^[18]^ It is important to note that the excessive activation of MCM/CMG helicases by Myc is not detrimental to ongoing DNA replication, and only produces shorter inter-origin distances due to more CMGs being active at one time.^[18–20]^ It is only when conditions create stalled replication forks that an excess of Myc and consequent CMG over-activation will make for an aberrant situation. From this perspective, whereas Myc is the primary driver of tumorigenesis, Myc creates a second tumordriver by its actions: deficient pools of reserve MCMs that reduce the efficiency of recovery from stalled fork conditions, thereby promoting tumorigenesis due to increased DNA damage and genomic instability.

## 4. Mismanagement of CMG Reserves by Myc Defines an Exploitable Vulnerability in Cancer

**Figure 2.**
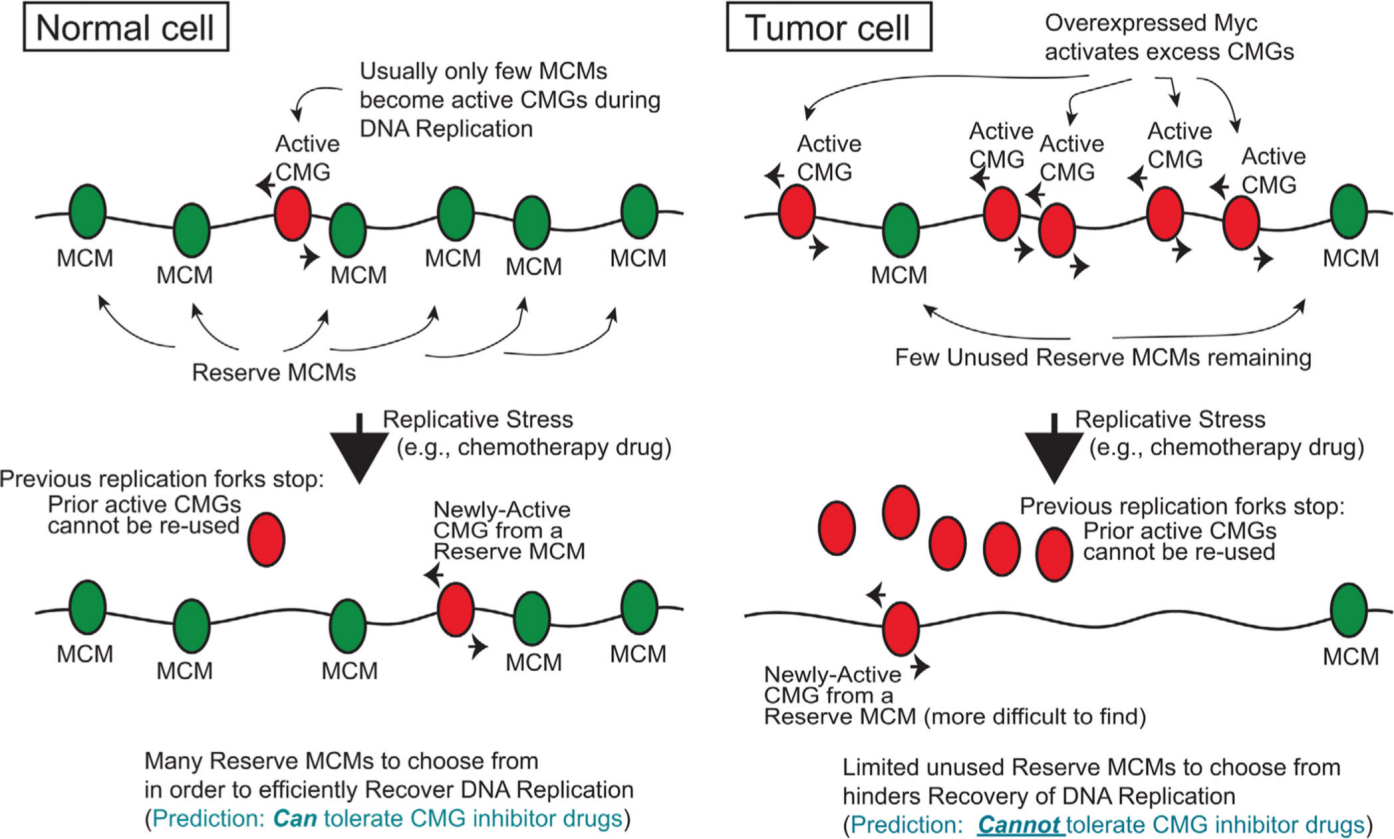
Tumor cells lack unused reserve MCMs/CMGs due to myc overexpression. (Each MCM/CMG shown represents a pair, for ease of display).

Thus far, the over-activation of reserve MCMs/CMGs by Myc has been illustrated as a potential strength of the process that selects for malignant outgrowths, in that loss of reserve MCM capacity due to Myc’s actions produces an unstable genomic environment that drives tumor formation and tumor heterogeneity. However, the same MCM-debilitating process that can drive tumorigenesis is likely to also be an *Achilles’ Heel* for a tumor, a Myc-driven weakness, and vulnerability. For example, in tumors with overexpressed Myc, the reserve MCM capacity may be reduced such that these tumors will display heightened sensitivity to chemical inhibitors that cause DNA replication fork stalling^[18]^ (Figure 2). Indeed, the clinical arsenal contains many forkstalling chemotherapeutic drugs, and there are examples of Myc-elevated tumor conditions that are more sensitive to such drugs.^[37,43]^ Given that Myc is overexpressed or deregulated in the vast majority of human malignancies, such a concept extends to a large number of neoplasms. In fact, it may be that one reason many chemotherapy drugs, including alkylating, crosslinking, and intercalating agents, and drugs that block nucleotide synthesis or topoisomerases, are effective in cancer management is due to tumor-specific conditions (e.g., Myc deregulation) in which recovery from fork-stalling drugs is impaired as a result of lowered reserve MCM/CMG capacity. We define this concept as the “reserve CMG helicase vulnerability.”

A more intriguing concept based on this vulnerability in reserve MCM mismanagement due to Myc overexpression is that small chemical inhibitors against the CMG enzyme itself (CMGi) may prove useful in clinical approaches. If the MCM/CMG reserves are already debilitated due to Myc’s actions, then administering a CMGi has the propensity to make a bad situation worse, further inactivating the remaining (now limited) reserve CMG helicases and likely increasing the sensitization of tumor cells to fork stalling chemotherapy (Figure 2). Importantly, because nontumor cells have not abrogated reserve MCM/CMG function due to anonco gene such as Myc, they would be predicted to display resistance to CMGi relative to the tumor cells, thus defining a therapeutic window for CMGi use. In essence, while a small reserve MCM deficiency can drive tumorigenesis, further loss of already reduced reserve MCM capacity with a CMGi may be lethal to a tumor cell and therapeutic. Such a concept can be tested experimentally in pre-clinical approaches where Myc-elevated cells are directly compared to Myc-normal cells, or by using siRNA-mediated methods that reduce existing high levels of Myc, followed by examination of these cell type differences for manipulated Myc expression on sensitivity to chemotherapy drugs.

In support of these concepts, it has already been demonstrated that future CMGi are indeed likely to be effective against tumor cells in a selective manner, and sensitize tumor cells to chemotherapeutic drugs.^[8]^ As discussed above, pancreatic ductal adenocarcinoma and colorectal cancer cells can be sensitized to clinically relevant chemotherapeutic (fork-stalling) drugs when the reserve complement of MCMs is depleted using siRNA-mediated methods, mimicking the presence of a future CMGi as a proof-of-principle.^[8]^ Mechanistically, this sensitization derives from the fact that tumor cells with experimental MCM reserve loss have a reduced ability to recover DNA replication after replicative stresses induced by exposure to fork-stalling chemotherapy drugs.^[8]^ More importantly, under similar conditions of reserve MCM debilitation by siRNA, non-tumor cells are less sensitive to fork-stalling drugs than tumor cells.^[8]^ This indicates that tumor cells are selectively compromised for further loss of reserve MCM capacity during replicative stress, and that a therapeutic window likely does exist for CMGi intervention in cancer treatment. It is note worthy here that the genetic basis (e.g., Myc or other oncogenes) for why the PDAC and CRC tumor lines tested in this study are vulnerable to CMG suppression is not known.^[8]^ Nonetheless, such studies and the potential for Myc to create a tumor-selective reserve MCM vulnerability in many human neoplasms strongly suggest that CMGi intervention may offer an innovative means of managing cancer in the clinic. We refer to this concept as the “reserve CMGi clinical hypothesis.”

## 5. The CMG Helicase as a Feasible Clinical Target for Cancer Intervention

A recent clinical trial found that pancreatic cancer patients who carry BRCA mutations are more sensitive to platinum therapy and PARP inhibitors (PARPi), indicating as a proof-of-principle that suppression of two DNA repair/recovery pathways is clinically advantageous for therapies that include DNA damaging drugs.^[44]^ In this case, the DNA repair/recovery ability of the tumor cells was already debilitated due to BRCA inactivation prior to exposure to a second repair-limiting drug, the PARPi.^[44]^ Given the potential or demonstrated weaknesses in the reserve MCM capacity of tumor cells that also limits repair/recovery, it becomes feasible to consider how a CMGi may be useful in similar future clinical regimens. For example, can a CMGi further debilitate a pre-existing weakness in MCM reserves and enhance chemotherapy for tumors carrying DNA repair deficiencies such as BRCA loss? Can a CMGi synergize with PARPi and DNA damaging drugs, or replace the PARPi drug in the clinical regimen? Do tumors with overexpressed Myc and debilitated MCM reserves provide a signature that predicts better responses to PARPi or other repair-limiting drugs in combination with fork-stalling chemotherapy? Can this be further enhanced with a CMGi included in patient treatment, concurrently or with different scheduling?

There are additional clinical considerations that justify anticancer intervention with a focus on targeting the CMG helicase. Recalcitrant metastatic lesions that fail to respond to conventional or personalized drug regimens due to biological changes from the primary lesions may yet retain MCM reserve vulnerabilities due to overexpressed Myc that sensitizes the distal lesions to CMGi. Likewise, tumors that fail first-line therapies may be suitable for second-line approaches involving CMGi due to genetic conditions such as Myc deregulation that affect MCM management. Examples of the latter include the *Tp53* and *RB1* deficient malignancies small cell lung cancer and osteosarcoma.^[34,45]^ These and other possibilities argue for the need to develop drugs that can effectively and specifically inhibit the CMG helicase. However, it should be noted that, since loss of reserve MCM/CMG capacity has the potential to be a tumordriver, chronic CMGi exposure in the clinic might promote secondary malignant growths. As with other chemotherapy drugs that can similarly cause secondary neoplasms, this would need to be factored into the design of clinical regimens in treating patients.

For clinical utility, the CMG helicase needs to be amenable to drug targeting with small chemical moieties, and preferably in a unique manner that is not easily overcome through biological changes in tumor cells. One way to target the CMG is through an indirect approach, by inhibiting druggable enzymes that are required for CMG assembly or activation. For example, Cdc7 is a kinase required for CMG assembly, and several groups have developed Cdc7i that are in early clinical trials (see Box 2). However, off-target effects of kinase inhibitors for Cdc7 may limit the effectiveness of such drugs. As such, the development of direct and specific CMG inhibitors may offer unique anticancer potential.

Although the Cdc45 and GINS subunits are not enzymatically active in terms of targetable clefts, the hexameric MCM ring of the CMG has six targetable ATP-binding and hydrolyzing domains^[6,7,11,46,47]^ (see Figure 1). These ATPase regions are likely biochemically unique from other ATPases such as kinases, since ATP binding and hydrolysis occurs between adjacent subunits of the MCM ring.^[7,11,47]^ For example, the Mcm3 subunit, adjacent to Mcm7, provides a necessary arginine residue to complete the ATP binding pocket with the canonical GxxGxGKs/t (GKs/t substituted with AKS or SKS residues in HsMCM2–7) motif on Mcm7 important for ribose binding, phosphate interactions, and ATP hydrolysis.^[7,11]^ Development of inhibitors against any of the ATPase clefts of the CMG will render the entire helicase inactive based on multiple studies of related CMG helicases in eukaryotes.^[7,11,46,47]^ Such inhibitors will also be able to target not only functional CMG helicases, but also non-functional dormant MCM hexamers (reserves). Toward this end, there is a report of successful inhibition in vitro of the yeast MCM complex using fluoroquinolone based drugs. However, the dosages used were very high and the ability of these compounds to inhibit the purified hCMG enzyme has yet to be determined experimentally.^[48]^

The CMG helicase is the primary replicative helicase used for DNA replication within replisomes, and the CMG has no redundant functional counterpart. Therefore, it is difficult to envision a clinical situation where tumors can circumvent the CMG helicase (and a CMGi) by using an alternative enzyme or pathway. While it is possible that mutations could be selected for in tumors that limit the binding of CMGi to a particular cleft (as often happens with some kinases), this would only be achievable if the mutation does not render the helicase debilitated. Mutated and weakened CMGs are not likely to allow survival of tumor cells that already have MCM/CMG reserve deficiencies. In fact, the latter concept could be tested using a combination of catalytically-mutant MCM subunit expression simultaneously with siRNA-mediated targeting of a separate MCM subunit that achieves overall reduction in MCM hexamer numbers.

## 6. Is Myc the Only Influence Over Reserve MCM Function?

**Figure 3.**
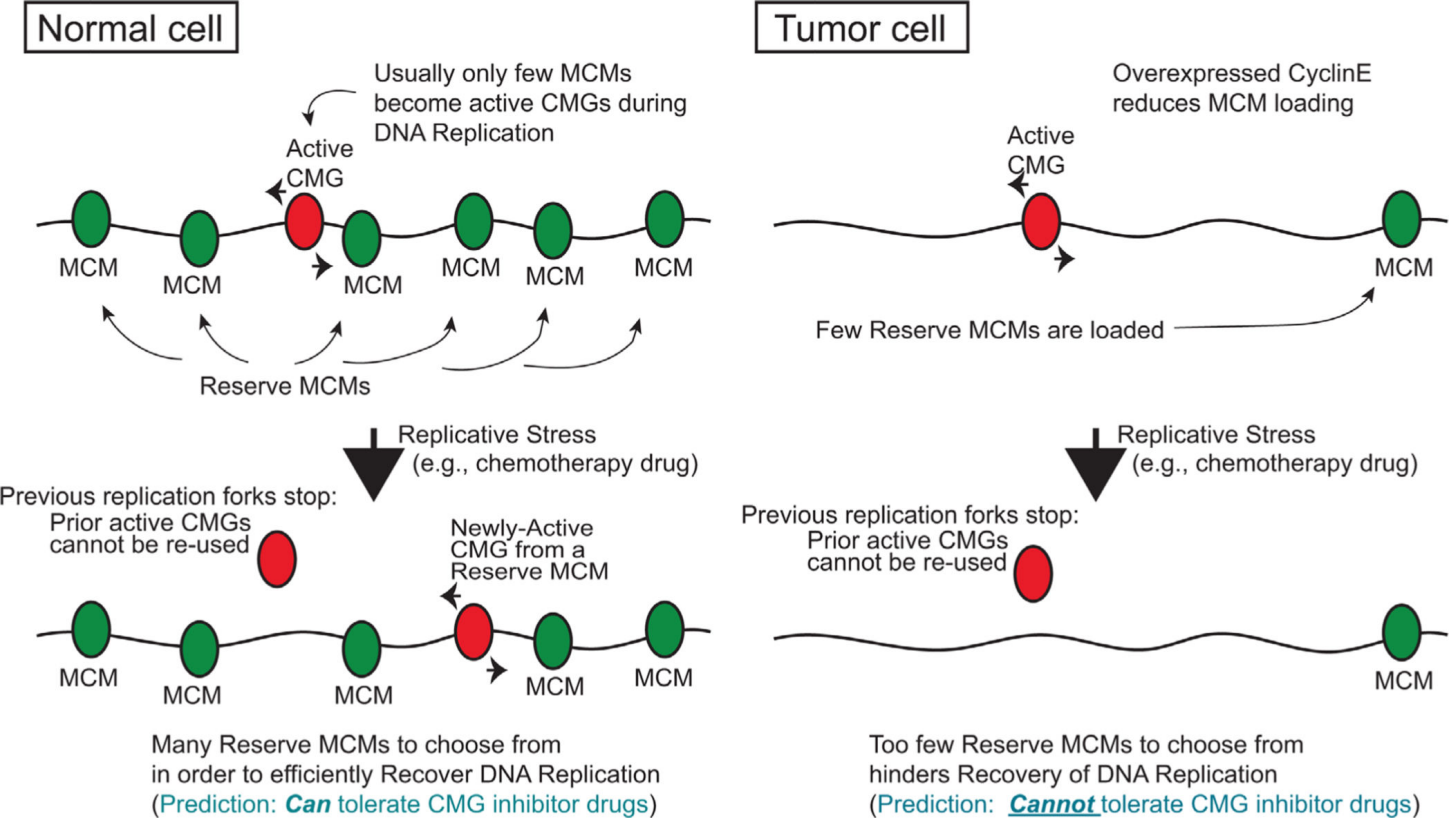
Tumor cells lack reserve MCMs/CMGs due to Cyclin E overexpression. (Each MCM/CMG shown represents a pair, for ease of display).

Much of this discussion has used the ability of Myc to mismanage the reserve MCM pool as a basis for explaining mechanisms of CMG control and consequent vulnerabilities for tumor cells. Beyond Myc, other oncogenes are likely to elicit MCM mismanagement. Cyclin E overexpression is present in several tumor types,^[49,50]^ is upregulated by Myc,^[51]^ and Cyclin E deregulation has been shown to be associated with acute onset of genomic instability.^[50,52,53]^ Interestingly, overexpression of Cyclin E causes a diminishing of the number of MCM hexamers that load onto chromatin/DNA prior to S-phase^[50]^ (Figure 3). Similar in outcome to Myc elevation, this results in an overall functionally decreased reserve MCM pool, but due to a reduction of total reserve MCMs that are present in S-phase versus over-activation of MCMs/CMGs by Myc that reduces the number of unused reserve MCMs (compare Myc effects in Figure 2 to Cyclin E effects in Figure 3).

Perhaps one explanation for why overexpressed Cyclin E has this effect on the reserve MCM pool derives from its ability to cause premature S-phase entry,^[52]^ which may force S-phase to begin before the full necessary complement of MCMs are loaded. Compounding this, Cyclin E overexpression was also found to promote abnormal origin firing within genes that are normally transcriptionally active and lack origin firing.^[52]^ This results in collapsed forks and DNA double strand breaks due to conflicts with the transcription apparatus.^[52]^ These effects, along with break-induced repair of damaged forks,^[54]^ appear to contribute to genome duplication and chromosomal rearrangements prevalent in cancers.^[52,54]^ In fact, overexpressed Myc was also found to have similar effects as Cyclin E on origin usage following premature S-phase entry.^[52]^ These effects of Cyclin E and Myc overexpression on diminishing MCM reserves and replication fork mismanagement thus generate DNA damage and genomic instability, which drives tumor promoting conditions,^[50,52–54]^ yet may also provide for sensitivity to CMGi and fork-stalling chemotherapy in the clinic (Figures 2 and 3). This would suggest that, minimally, there are three established means to mismanage MCM reserves: i) mutation of *Mcm* genes, ii) Myc-driven over-activation of reserve MCMs, and iii) Cyclin E-driven reduction of MCM availability. Added to this are other effects of Myc and Cyclin E on creating irregular replication fork dynamics due to premature S-phase entry.

Whether and how other oncogenes affect and/or mismanage reserve MCM pools remains to be determined. An elegant review of oncogene-induced replicative stress describes many cellular mechanisms that oncogenes influence leading to replicative stress as an outcome,^[55]^ suggesting the likelihood that other oncogenes may indeed also affect the dynamics of the reserve MCM pool, directly or indirectly. Or perhaps pathways not typically associated with oncogenic deregulation may be found to mismanage reserve MCM pools and thus act as drivers of carcinogenesis. Notably, tumors such as sarcomas often lack a specific oncogenic driver as an explanation for their development. Instead, some sarcomas (e.g., osteosarcomas) contain a large number of genomic rearrangements, chromosome losses, an euploidy, and hyper diploidy. Although a specific molecular reason for why these genomic issues arise is not known, one possibility is that the reserve MCM pool has been adversely affected at some stage in tumor development, rendering the genome unstable and causing selection for sarcoma outgrowth. Such a hypothesis might be welcome not only from a mechanistic point of view in terms of why such sarcomas develop, but also from a clinical translational perspective in which the mismanaged CMG reserves become a viable target in sarcomas for chemotherapeutic intervention due to predicted weaknesses in this process.

## 7. Conclusions and Outlook

The ability of Myc to over-stimulate conversion of MCMs into functional CMG helicases necessarily draws on the pool of reserve MCMs that could become active CMGs in times of need, such as during replicative stresses that occur normally, by oncogenes, or through insult with chemotherapy. This role for Myc in CMG (mis)management clearly provides at least one explanation for the loss of genome integrity that is common to many tumor types, and at the same time can explain how tumors can evolve under diminished reserve MCM capacity into heterogenous mixtures of cells at the genomic and physiologic levels. Interestingly, such a hypothesis for Myc would also suggest that this CMG deregulation would likely create a loss of viability for tumor cells as they evolve, due to stochastically acquired genomic issues that are not compatible with survival.

How might tumor cells evolve to override loss in CMG control by Myc and produce surviving cells? The CMG helicase is not a self-contained enzyme working in isolation, but rather is part of a large complex machine called a replisome, which incorporates proteins such as DNA polymerases, topoisomerases, cofactors, and other subunits.^[56]^ Perhaps as a result of Myc-driven genomic instability/changes and selection pressure, some of these associated proteins will be found in future experiments to be deregulated in tumor cells, compensating for deficiencies in CMG function and further creating tumor heterogeneity in the surviving cells. However, even in the presence of such overriding events, the fundamental issue will remain that CMG fidelity has been weakened in tumors and remains an exploitable vulnerability due to the tumor’s need for a minimal CMG functionality to survive. Clearly, the future will uncover answers to these questions, which have important implications not only for our basic understanding of tumor evolution, but also for identifying other exploitable vulnerabilities.

## Information Box 1: Myc-Driven CMG Assembly in Mammalian Cells Requires Recruitment of Chromatin Modifying Complexes

Using an innovative experimental system in Chinese hamster ovary (CHO) cells that allows one to observe higher order chromatin unfolding at specific sites *in vivo*, it was found that targeting Myc (via LacI-LacO interactions) to such sites caused a localized assembly and activation of CMG helicases via the recruitment of Cdc45 and GINS to pre-established MCM complexes ^[20]^. This CMG stimulation by Myc was dependent on the creation of chromatin accessibility at targeted sites. The effect was specific for Myc, as other transcription factors that similarly cause chromatin unfolding could not promote CMG assembly. Two histone acetyl-transferases (HATs) known to interact with Myc were required for this, specifically Tip60 and GCN5. TRRAP, which mediates HAT interactions with Myc, was also required for Myc to induce CMG activation at targeted sites. Consistent with these observations, the Myc Box II domain (MB-II) of Myc, which is required for the transforming ability of Myc and for efficient interactions with these HAT/TRRAP complexes, was required for CMG activation at targeted sites. Intriguingly, overexpression of GCN5 or Tip60 with mutant Myc lacking MB-II could rescue CMG assembly and activation.

These observations were also recapitulated in mouse keratinocytes expressing regulatable MycER, where induction of the intact MycER protein recruited GCN5 and Tip60, and caused CMG assembly and activation at MycER-bound chromosomal sites *in vivo*. MycER also stimulated excessive levels of DNA replication in the mouse cells. In contrast, mutant MycER lacking MB-II was again defective for CMG assembly and stimulation of DNA replication in the keratinocytes. Importantly, endogenous Myc was shown to be required specifically in the late-G1 interval for CMG assembly, and Myc’s loss could be rescued by induction of MycER, but not mutant MycER lacking MB-II. Endogenous Myc and activated-MycER both interact with Cdc45 in late-G1, and Cdc45 interactions with chromatin and MCM complexes in late-G1 require intact Myc and GCN5 function. Finally, using a human keratinocyte line (HaCaT), it was shown that Myc is required for chromatin accessibility at the Lamin B2 DNA replication origin in late-G1, and that the function of GCN5 is required to maintain chromatin accessibility at this origin. These results provide a mechanistic understanding of how Myc can directly promote CMG assembly and activation in mammalian cells via changes in chromatin accessibility that are separate from, but related to, transcriptional functions of Myc.

## Information Box 2: Cdc7 Kinase Inhibitors as an Indirect Means to Target the hCMG in the Clinic

CMG assembly and activation are regulated in part by the action of Cdc7 in association with a subunit called Dbf4 ^[57]^. Cdc7-Dbf4 and Cdk2 kinases phosphorylate components of the MCM complex, thereby allowing interactions between MCM, Cdc45, and GINS subunits to form the CMG helicase ^[58]^. Cdc7 also forms complexes with another regulatory subunit called Drf1 ^[59]^. Beyond its role in CMG assembly, Cdc7 also appears to have a role in activating the S-phase checkpoint in response to inhibitors of DNA replication ^[60]^. Cdc7 is elevated in tumor cells and tissue, perhaps due to the proliferative status of tumor tissue and the need for Cdc7 in S-phase, but has not been found to be mutated or amplified in human malignancies [^[61]^ and references therein]. Inhibition of Cdc7 in tumor cells elicits an apoptotic response, whereas in non-tumor cells a cell cycle checkpoint arrest occurs without apoptosis ^[61]^. These characteristics of Cdc7 are the basis for the development of Cdc7 inhibitors (Cdc7i) for translational use in the cancer clinic. ATP-competitive small molecule inhibitors have been identified and entered Phase 1/2 trials by several companies and investigators, including Nerviano Medical Sciences (NMS-1116354), Bristol-Myers Squibb (BMS-863233), Sierra Oncology (SRA141), Takeda (TAK-931), others from Novartis and Sanofi-Aventis, and a dual Cdc7i/Cdk9i inhibitor called PHA-767491 ^[61–63]^. While pre-clinical results for Cdc7i are promising in their tumor specificity and effectiveness, the success or limitation of these Cdc7i in the clinic awaits the outcome of these and other trials. For further mechanistic details on the development of Cdc7i for anti-cancer therapeutics, the reader is referred to an elegant review by Colotta and colleagues ^[61]^.

From the perspective of inhibiting the reserve hCMG helicases, the indirect targeting of the hCMG with Cdc7i presents advantages to test the hypotheses put forth herein, given the head start these drugs have at the moment. Success with Cdc7i in the clinic may be attributable, in part, to the indirect targeting of hCMG functionality that is potentially diminished in cancers as a result of oncogenic mismanagement. However, some important caveats for Cdc7i clinical interpretations must be considered. As is inherent to many kinase inhibitors, current Cdc7i also target other kinases, examples including Pim1, Cdk9, GSK3α, and GSKß ^[61, 62, 64]^. Clinically speaking, such off-target effects may be considered advantages for suppressing secondary pathways important in cancer cells, thereby enhancing the clinical efficacy of such Cdc7i drugs ^[62, 64]^. However, the fact that these Cdc7i are not specific for Cdc7 makes it difficult to conclusively interpret the effects of Cdc7i as being derived in whole or part from indirect hCMG suppression. Also, since Cdc7 has other roles besides hCMG regulation, the question of whether direct hCMG inhibition offers unique advantages for anti-cancer therapeutics will remain unknown from these Cdc7i trials.
